# Establishment of an Apical-Out Organoid Model for Directly Assessing the Function of Postbiotics

**DOI:** 10.4014/jmb.2405.05034

**Published:** 2024-09-09

**Authors:** Yeonoh Cho, Moon-Hee Sung, Hee-Taik Kang, Jong Hun Lee

**Affiliations:** 1 Department of Food Science and Biotechnology, College of Bio-Nano Technology, Gachon University, Gyeonggi 13120, Republic of Korea; 2 KookminBio Corporation, Seoul 02826, Republic of Korea; 3 Department of Family Medicine, Severance Hospital, Yonsei University College of Medicine, Seoul 03722, Republic of Korea

**Keywords:** Apical-out organoid, postbiotics, tight junction, mitochondria homeostasis

## Abstract

In vitro organoids that mimic the physiological properties of in vivo organs based on three-dimensional cell cultures overcome the limitations of two-dimensional culture systems. However, because the lumen of a typical intestinal organoid is internal, we used an apical-out intestinal organoid model in which the lumen that absorbs nutrients is outside to directly assess the function of postbiotics. A composite culture supernatant of *Lactiplantibacillus plantarum* KM2 and *Bacillus velezensis* KMU01 was used as a postbiotic treatment. Expression of COX-2 decreased in apical-out organoids co-treated with a lipopolysaccharide (LPS) and postbiotics. Expression of tight-junction markers such as ZO-1, claudin, and Occludin increased, and expression of mitochondrial homeostasis factors such as PINK1, parkin, and PGC1a also increased. As a result, small and large intestine organoids treated with postbiotics protected tight junctions from LPS-induced damage and maintained mitochondrial homeostasis through mitophagy and mitochondrial biogenesis. This suggests that an apical-out intestinal organoid model can confirm the function of food ingredients.

## Introduction

The intestinal mucosal barrier regulates the absorption of essential nutrients, prevents the entry of harmful contents, and maintains balance. Intracellular junctions of epithelial cells are critical to managing the properties of the mucosal barrier [1]. Among the junctions that make up the intestinal barrier, tight junctions play a pivotal role in maintaining barrier integrity. Tight junctions comprise proteins such as Occludin, claudin, and zonula occludens (ZO) [2]. Lipopolysaccharide (LPS) treatment has been demonstrated to induce permeability of the intestinal mucosal barrier by disrupting tight-junction structures and the secretion of cytokines and inflammatory mediators [3].

Mitochondria are organelles that play important roles in maintaining cellular homeostasis and act as an integral part of stress-sensing mechanisms [4]. Mitochondrial dysfunction contributes to the development of gut diseases [5]. Additionally, the mitochondrial production factor peroxisome proliferator–activated gamma coactivator 1-alpha (PGC1α) helps maintain mitochondrial integrity, strengthens intestinal barrier function, and reduces inflammation [6]. Maintaining mitochondrial balance is therefore essential. Mechanisms that maintain mitochondrial balance include biogenesis and mitophagy. Mitophagy is a selective form of autophagy that removes damaged or unnecessary mitochondria from cells and maintains mitochondrial homeostasis by regulating mitochondrial contents and metabolism along with mitochondrial biogenesis [7, 8].

Postbiotics include all substances released by microorganisms or produced through metabolic activities and are characterized by diverse effects, sources, and composition [9]. Postbiotics have beneficial effects on hosts, either directly or indirectly. In addition, postbiotics are considered superior models for understanding the complex interactions that occur continuously within our bodies compared with probiotics, and research is being conducted on their effects on our bodies [10].

Organoids are stem cell–derived, autologous, three-dimensional cultures that express, to some extent, the composition, structure, and function of cell types from other tissues [11]. They resemble a variety of cell types found in the human body and have the potential to complement existing model systems and extend basic biological research, medical research, and drug discovery to the physiologically relevant human environment [12]. However, research on organoids in food supplies is lacking. Intestinal organoids form a closed system and the lumen is inside the round-shaped organoids. When evaluating the absorption of food and the performance of ingredients, injecting food ingredients into organoids through microinjection can be difficult. However, if the polarity of existing organoids is reversed, the apical side of the lumen would be accessible on the outside [13]. We therefore designed an experiment using “apical-out” organoids that overcome the limitations of regular organoids. In this paper, we describe treating intestinal organoids with postbiotics to protect intestines from inflammation caused by LPS and improve intestinal barrier function. We hypothesized that postbiotics can maintain intestinal homeostasis and tested the hypothesis using an apical-out organoid-based experimental model.

## Materials and Methods

### Organoid Fabrication

In this experiment, 6-week-old C57BL/6 male mice purchased from Orientbio (Orientbio, Republic of Korea) were sacrificed. All animals were treated humanely following criteria outlined in the Guide for the Care and Use of Laboratory Animals, prepared by the National Academy of Science and published by the National Institutes of Health. All experiments were approved by the Institutional Animal Care and Use Committee (IACUC 190190) of CHA University. Small intestines and colons were cut into pieces 0.5–1 cm in length. The pieces were collected in a 50 ml conical tube and then washed with a phosphate-buffered saline (PBS) solution. Intestines and colon crypts were extracted from small pieces with 2 mM EDTA and 20 mM EDTA, respectively. After the cells are sufficiently separated, the cells were filter through a 70 μm cell strainer. The crypts collected in the tube were embedded in Matrigel gel, seeded in 24-well plates, and incubated for 15 min at 37°C. A complete culture medium containing various growth factors (with Y-27632) was added to each well after the Matrigel gel solidified. The complete culture medium for small intestine and colon organoids contained 25% and 50% noggin-conditioned medium, respectively. A 10% R-spondin conditioned medium, 10 mM HEPES, 1 mM N-acetyl-L-cysteine, 50 ng/ml EGF, and 10 μM y-27632 were also added to prepare the complete culture medium. The culture medium (without Y-27632) was exchanged every 2 or 3 days.

### Preparation of Postbiotics

*Lactiplantibacillus plantarum* KM2 (*L. plantarum* KM2) and *Bacillus velezensis* KMU01 (*B. velezensis* KMU01), isolated and identified from traditional Korean fermented foods, were utilized after genomic analysis [14, 15]. *L. plantarum* KM2 and *B. celezensis* KMU01 were each inoculated into MRS and TSA broth media at 1% (v/v) and cultured at 37°C for 24 h until the number of cells reached 109 CFU/ml. The postbiotics culture supernatant was obtained by centrifuging at 10,000 ×*g* for 30 min at 4°C to remove the bacterial cells, and each separated culture supernatant was subsequently filtered through a 0.22 μm syringe filter. Finally, the cell-free supernatants of the probiotics were mixed in the following ratio to be used as the research sample: *L. plantarum* KM2 to *B. celezensis* KMU01 at a ratio of 2:1.

### Apical-Out Organoid Formation and Treatment

The medium was removed, and organoids embedded in Matrigel were collected with PBS in a 15 ml conical tube and subcultured by TrypLE Express (Gibco, USA). The organoids were incubated at 37°C for 5 min, centrifuged at 800 *g* for 5 min, and washed with ice-cold PBS. The organoids were reseeded in a 24-well cell-floater plate (SPL, Republic of Korea) and cultured for 3 days. Next, 10 μM of LPS, and 1% and 5% cell-free supernatant postbiotics were added to well plate. The cell-free supernatant postbiotics were supplied by Professor Moon-hee Sung (Kookmin University). The postbiotics were prepared by mixing the culture supernatant of *L. plantarum* and *B. velezensis*. The organoids were harvested 2 h after treatment.

### Immunofluorescence

Basal and apical-out organoids were collected and fixed with 4% formaldehyde for 20 min at room temperature. Next, 5% normal goat serum and 0.1% Triton X-100 were added to PBS and incubated for 30 min at room temperature to block non-specific binding and allow for permeabilization. The primary antibodies, rabbit anti–ZO-1 (Abcam, UK), and mouse β-catenin (Santa Cruz, USA), were diluted to 1:500 and 1:200, respectively. The organoids with primary antibodies were incubated overnight at 4°C. The organoids were washed with PBS, and the secondary antibodies, goat anti-rabbit immunoglobin G (IgG) Alexa Fluor 488 (Abcam) and goat anti-mouse IgG Alexa Fluor 568 (Invitrogen, USA), were diluted at 1:1000 and incubated for 1 h at room temperature. The organoids were stained with DAPI and analyzed by confocal microscopy.

### Western Blotting

The organoids were lysed in NP40 (Invitrogen) and a protease inhibitor cocktail (Gendepot, USA). Equal amounts of protein were separated by gel electrophoresis and transferred to polyvinylidene difluoride membranes. The membranes were incubated for 1 h at room temperature in a blocking solution of 5% bovine serum albumin. The primary antibody, ZO-1 (Abcam), was diluted to 1:3000, and Occludin (Abcam), PINK1 (Santa Cruz), Parkin (Santa Cruz), PGC1-α (Santa Cruz) and β-actin (Santa Cruz) were diluted to 1:1000. The membranes were incubated with primary antibodies at 4°C, overnight. After three washes with TBST, the membrane was allowed to react with horseradish peroxidase –conjugated anti-IgG (Promega, USA) and visualized using electrochemiluminescence. The protein ratios were calculated with ImageJ software.

### RNA Extraction and Real-Time Quantitative PCR

The RNA was extracted using TRIzol reagent (Invitrogen) and quantified with nanodrop. Total RNA was synthesized as cDNA for real-time quantitative PCR using TOPreal qPCR 2x PreMIX (Enzynomics, Republic of Korea). The relative expression mRNA levels were calculated using the ΔΔCt method with GAPDH as a housekeeping gene.

### Statistical Analysis

Data were expressed as the mean ± standard deviation (SD) and compared using a One-Way ANOVA. The results were considered statistically significant at *p* < 0.05. Experiments were performed at least three times.

## Results

### Fabrication of Mouse Basal and Apical-Out Intestinal Organoids

**Fig. 1:**
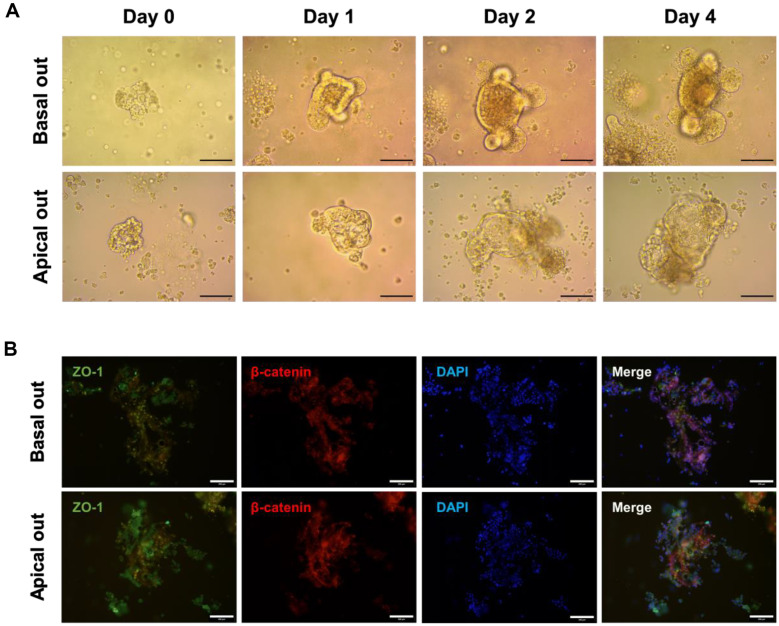
Morphological differences between basal-out and apical-out intestinal organoids. (**A**) The small intestine of a 7-week-old mouse was cultured in a Matrigel matrix containing organoid growth medium; the polarity was then reversed to form an apical-out organoid. We verified the formation of organoids (basal-out organoids) and polarity-inverted organoids (apical-out organoids) cultured in the Matrigel matrix. (**B**) Immunofluorescence image of basal and apical-out organoid. Organoid morphology was observed under a microscope 3-4 days after crypt seeding to check their reversal polarity. The organoids were stained with ZO-1, β-catenin, and DAPI to localize the apical side, basal side, and nucleus, respectively. Scale bars = 200 μm.

To establish a more effective model to test the bioactivity of postbiotics, basal-out mouse organoids were prepared from mouse intestines. Crypts were isolated from small parts of the mouse intestines. The isolated crypts were applied to Matrigel gel supplemented with an organoid growth medium. As the culture progressed, the organoids initiated from the crypts differentiated into larger forms, with villi and buds forming on day 2 (Fig. 1A). In general, most bioactive substances interacted with the apical membrane of intestinal epithelial cells and were absorbed into tissues through villi. However, conventional intestinal organoids cultured on Matrigel gel are enclosed systems with inward-facing villi. Traditional mouse intestinal organoids were therefore first cultured in Matrigel gel to expose the apical membrane of epithelial cells and then induced in a suspension culture system to reverse their polarity. We confirmed the structural difference between apical and basal-out organoids through immunofluorescence staining (Fig. 1B).

### Postbiotics Reduce LPS-Induced Inflammation in Apical-Out Organoids

**Fig. 2:**
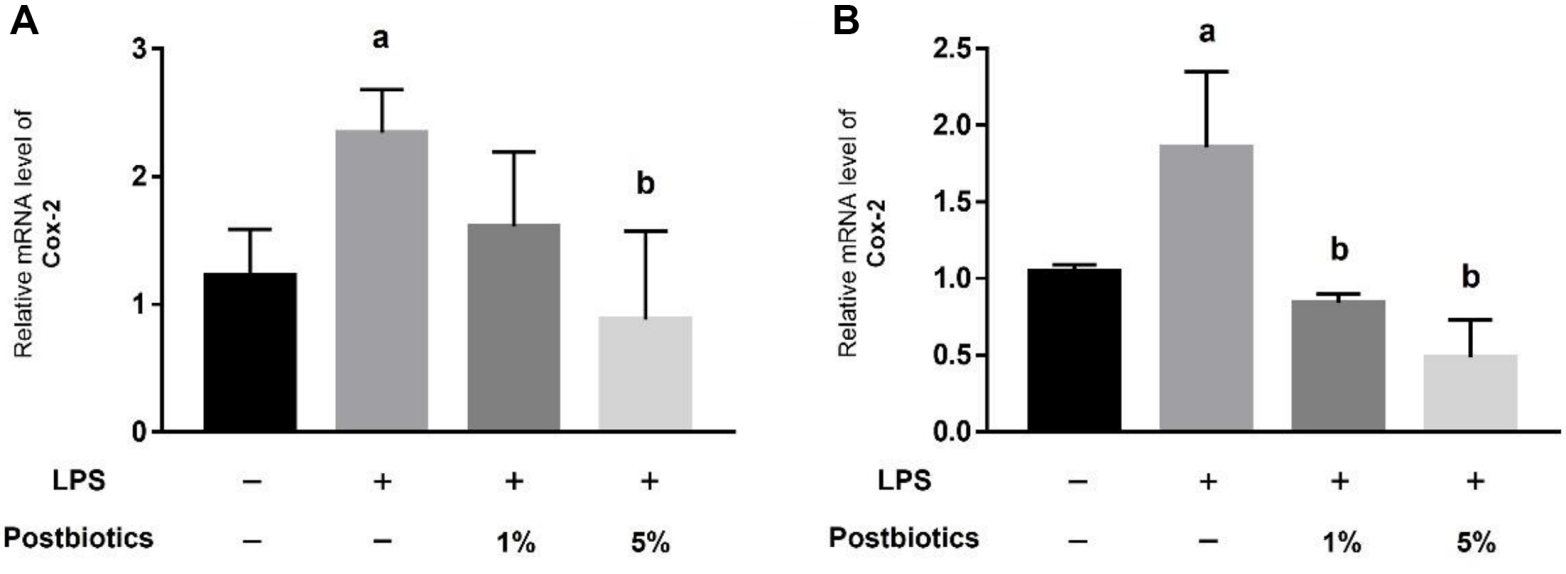
Relative inflammatory mRNA expression level in intestinal organoids. (**A**) Relative COX-2 mRNA expression levels in intestinal organoids. (**B**) Relative COX-2 mRNA expression levels in colon organoids. Each graph shows the relative value compared with that of a control. Bars represents the mean ± SD. Data represent the means of *n* = 3. Column a: *p* < 0.05 vs. control. Column b: *p* < 0.05 vs. LPS-treated group.

Damage was mimicked by treating apical-out organoids with LPS. When apical-out organoids from the small intestine were treated with LPS, the mRNA expression of COX-2, a representative inflammatory factor, increased. When treated with 5% postbiotics, expression decreased to a level similar to those of the control (Fig. 2A). Similarly, when apical-out colon organoids were treated with LPS, mRNA expression of COX-2 increased, and when treated with postbiotics, expression decreased compared with the LPS group (Fig. 2B). These results show that postbiotics have a preventive effect on intestinal inflammation in apical-out intestinal and colon organoids.

### Postbiotic Treatment Increases Tight Junctions in LPS-Induced Intestinal Organoids at the Protein Level

**Fig. 3:**
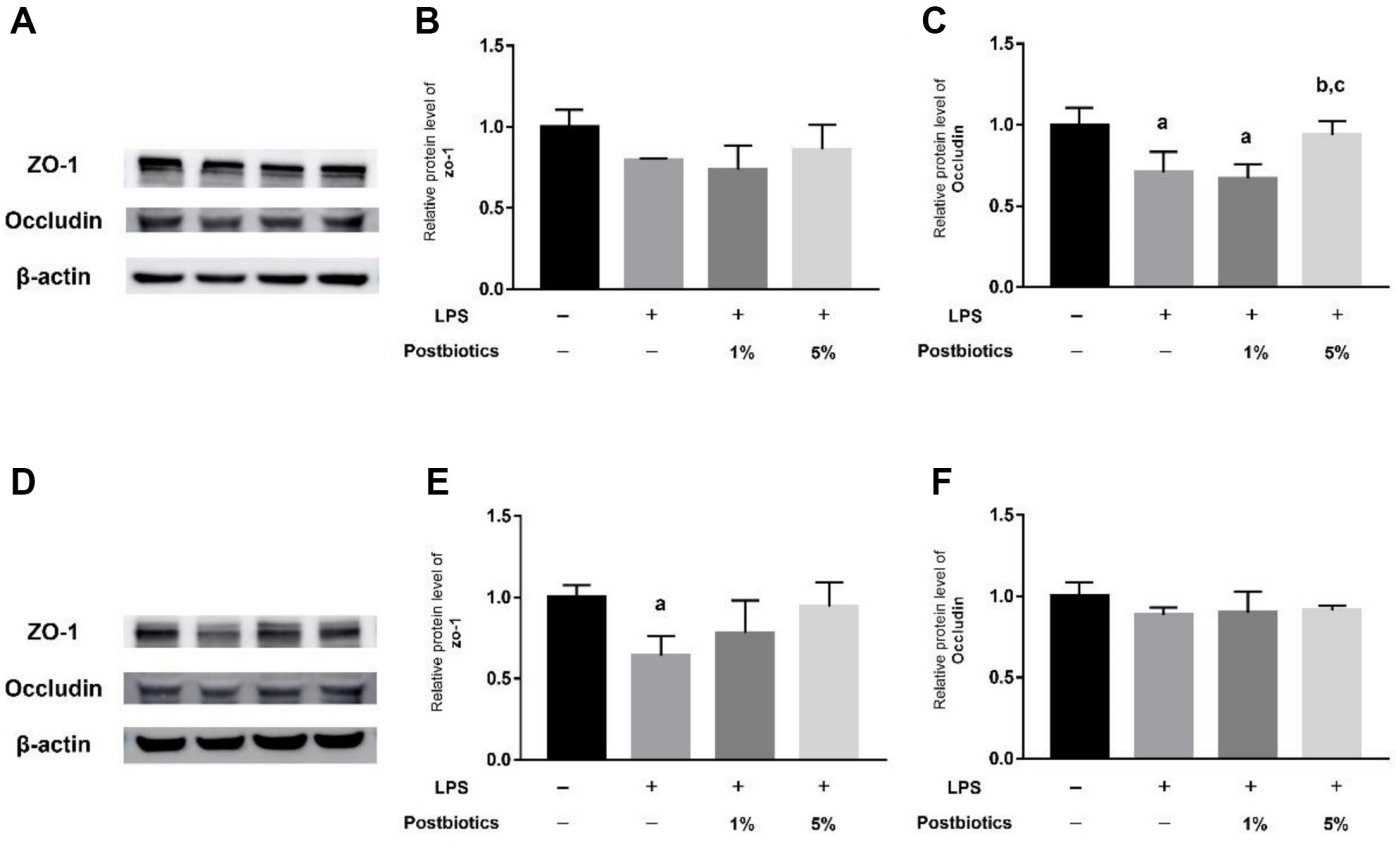
Protein expression of tight-junction markers in intestinal and colon organoids. (**A**) Western blotting of a tight-junction marker in intestinal organoids. (**B**) Relative protein expression of ZO-1 in intestinal organoids. (**C**) Relative protein expression of Occludin in intestinal organoids. (**D**) Western blotting of a tight-junction marker in colon organoids. (**E**) Relative protein expression of ZO-1 in colon organoids. (**F**) Relative protein expression of Occludin in colon organoids. Each graph shows the relative value compared with that of the control. Each bar represents the mean ± SD. Data represent the means of *n* = 3. Column a: *p* < 0.05 vs. control. Column b: *p* < 0.05 vs. LPS-treated group. Column c: *p* < 0.05 vs. 1% postbiotics group.

To observe the effect of postbiotics on intestinal epithelial tight junctions, organoids were treated with LPS to induce intestinal damage. Protein expression of ZO-1 and Occludin was confirmed in intestinal and colon organoids (Fig. 3). There was no difference in protein expression of ZO-1 in intestinal organoids. However, protein expression of Occludin decreased upon treatment with LPS but recovered to the control level upon treatment with 5% postbiotics (Fig. 3A–3C). In colon organoids, ZO-1 expression was significantly decreased in the LPS-treat group compared to the control group, and expression was similar to that of the control group when treated with 5% postbiotics. (Fig. 3D–3F).

### Postbiotics Increase Tight-Junction mRNA Expression in LPS-Treated Organoids

**Fig. 4:**
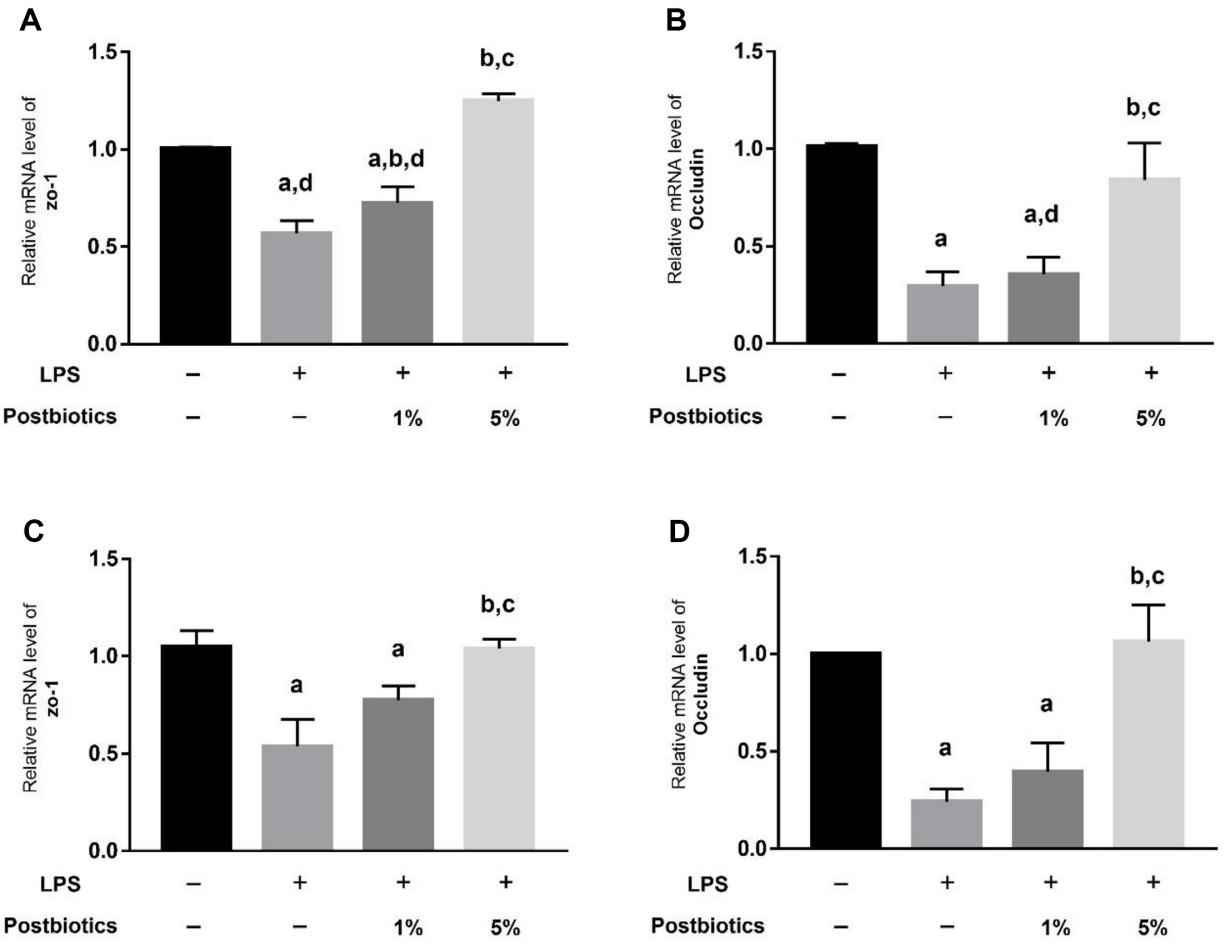
Relative mRNA expression levels of tight junctions in intestinal and colon organoids. (**A**) Relative mRNA level of ZO-1 in intestinal organoids. (**B**) Relative mRNA level of Occludin in intestinal organoids. (**C**) Relative mRNA level of ZO-1 in colon organoids. (**D**) Relative mRNA level of Occludin in colon organoids. Each graph shows the relative value compared with that of the control. Each bar represents the mean ± SD. Data represent the means of *n* = 3. Column a: *p* < 0.05 vs. control. Column b: *p* < 0.05 vs. LPS-treated group. Column c: *p* < 0.05 vs. 1% postbiotics group. Column d: *p* < 0.05 vs. 5% postbiotics group.

The expression of tight-junction markers was confirmed at the mRNA level when LPS-treated organoids were treated with postbiotics. mRNA expression of ZO-1 and Occludin decreased during LPS treatment in intestinal organoids and colon organoids. Expression did not recover when treated with 1% postbiotics, but treatment with 5% postbiotics did result in recovery of expression to a level similar to those of the control groups (Fig. 4A–4D).

### Postbiotic Treatment Maintains Mitochondrial Homeostasis in Organoids through Mitophagy and Mitochondrial Neogenesis

**Fig. 5:**
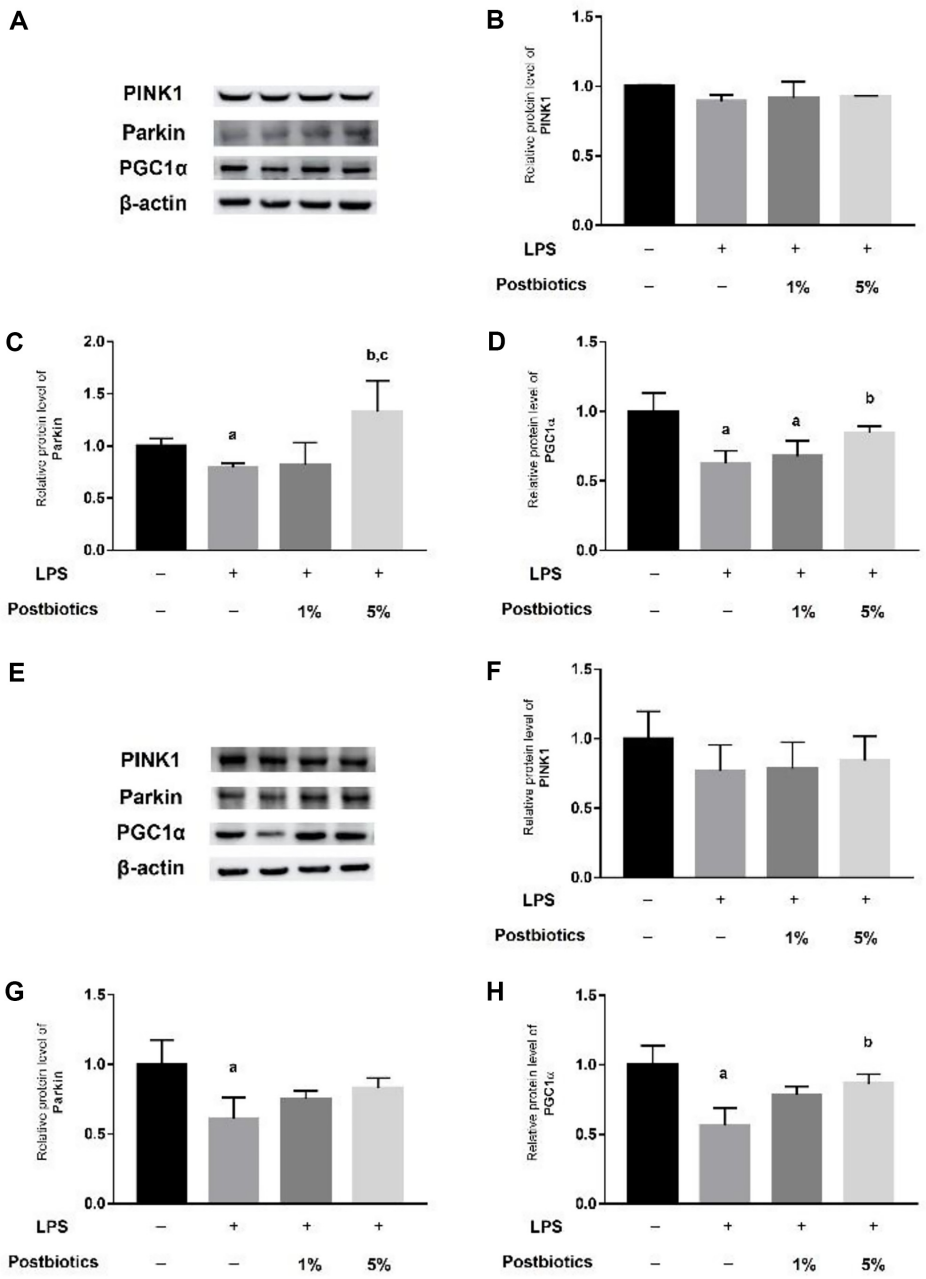
Relative protein expression level of mitochondria balance–related marker in intestinal and colon organoid. (**A**) Western blotting of mitochondria balance marker in intestinal organoids. (**B**) and (**C**) Relative protein levels of mitophagy markers PINK1 and Parkin, respectively, in intestinal organoids. (**D**) Relative protein level of PGC1α, a mitochondria biogenesis marker, in intestinal organoids. (**E**) Western blotting of mitochondria balances markers in colon organoids. (**F**) and (**G**) Relative protein levels of mitophagy markers PINK1 and Parkin, respectively, in colon organoids. (**H**) Relative protein level of PGC1α, a mitochondria biogenesis marker, in colon organoids. Each graph shows the relative value compared with that of the control. Each bar represents the mean ± SD. Data represent the means of *n* = 3. Column a: *p* < 0.05 vs. control. Column b: *p* < 0.05 vs. LPS-treated group. Column c: *p* < 0.05 vs. 1% postbiotics group.

In cells, mitochondria damaged by external stimuli are removed through a series of autophagy processes called mitophagy, and new mitochondria are created to maintain mitochondrial homeostasis [16]. Protein expression of Parkin, a representative factor of mitophagy, was decreased in LPS-treated intestinal organoids. However, when intestinal organoids were treated with 5% postbiotics, protein expression of Parkin significantly increased (Fig. 5A–5C). In addition, when treated with 5% postbiotics, protein expression of PGC1a, a mitochondrial production factor, increased significantly compared with that of the LPS-treated group (Fig. 5D). In colon organoids, which are similar to organoids found in the small intestine, the expression of mitophagy and mitochondrial production factors increased upon postbiotic treatment (Fig. 5E–5H).

## Discussion

In this study, the effects of postbiotics on protecting LPS-induced intestinal-barrier damage and mitochondrial balance were observed in apical-out intestinal organoids and colon organoids.

First, we confirmed the structure and establishment of apical-out organoids to create a proper apical-out organoid model (Fig. 1). As shown in Figure 1A, morphological differences between basal-out and apical-out organoids are not easily observed. Apical-out and basal-out organoids were revealed by staining for proteins expressed in different locations of epithelial cells (Fig. 1B). ZO-1 and beta-catenin form cell-cell junctions, but ZO-1 is a tight-junction protein, and beta-catenin is an adherens-junction protein. When comparing the locations of the tight and adherens junctions, the tight junction exists on the more apical side compared with the adherens junction [17]. It was confirmed that the protein expression of ZO-1, which exists on the apical side, was expressed more externally in apical-out organoids than in basal-out organoids (Fig. 1B). This indicates the formation of an apical-out organoid with polarity reversed from that of a basal-out organoid.

Proteins extracted from *L. delbrueckii* CIDCA 133 alleviated intestinal inflammation and colonic epithelial damage in a DSS-induced ulcerative colitis mouse model [18], and *L. casei* DG postbiotics reduced inflammatory cytokines in an irritable bowel syndrome model [19]. In this paper, when apical-out organoids from the small intestine and colon were treated with LPS, gene expression of COX-2 increased, and when treated with 5%postbiotics, gene expression decreased compared to LPS-treated group (Fig. 2A).

Postbiotics derived from *L. plantarum* 1.0386 improved tight-junction damage caused by LPS in caco-2 cells, and postbiotics from *L. rhamnosus* GG promote tight-junction protein expression and protect the intestinal barrier [20, 21]. In this paper, we confirmed changes in tight-junction gene and protein expression upon postbiotic treatment in apical-out organoids from the small intestine and colon. When treated with postbiotics in apical-out organoids from the small intestine, ZO-1 gene expression level and Occludin protein and gene expression increased (Fig. 3). In apical-out organoids from the colon treated with 5% postbiotics, ZO-1 protein and gene expression increased compared with an LPS-treated group, and gene expression of Occludin increased (Fig. 4).

Mitochondrial homeostasis is tightly regulated by fine coordination of two opposing processes: mitophagy, which removes damaged mitochondria; and the creation of new mitochondria by mitochondrial biogenesis [22]. When PINK1, which acts as a molecular sensor of damaged mitochondria accumulates on the mitochondrial surface, mitophagy, which removes depolarized mitochondria, is stimulated [23]. PINK1 activates parkin by directly phosphorylating the similar S65 residue of ubiquitin and S65 in the UbI domain of parkin [24]. PGC1α, a central regulator of mitochondrial biogenesis, activates mitochondrial transcription factors, which increase the expression of nuclear-encoded mitochondrial proteins, bind to target mtDNA, and activate mitochondrial transcription and replication [25]. As shown in Fig. 5, the expression of parkin increased upon postbiotics treatment compared to the LPS-treated group, indicating that mitophagy was stimulated and initiated. In addition, it was confirmed that postbiotic treatment increased mitochondria biogenesis through increased expression of PGC1a. The results depicted in Fig. 5 confirm that postbiotic treatment maintained mitochondrial homeostasis by increasing mitophagy and mitochondria biogenesis.

In conclusion, postbiotic treatment reduces LPS-induced inflammation in apical-out intestinal organoids and protects tight-junction damage. It also improves mitophagy and mitochondrial biogenesis and maintains mitochondria homeostasis by removing damaged mitochondria and creating new mitochondria.

## Figures and Tables

**Table 1:** Primer sequence for qRT-PCR.

		Primer sequence
GAPDH	F	5'-AACAGCAACTCCCACTCTTC-3'
	R	5'-GAGTAAGAAACCCTGGACCAC-3'
Cox-2	F	5'-CCAGATGCTATCTTTGGGGA-3'
	R	5'-CTCAATACTGGAAGCCGAGC-3'
ZO-1	F	5'- ACAGGCCATTACGAGCCTCT -3'
	R	5'- GGAGGCTGTGGTTTGGTAGC -3'
Occludin	F	5'- CTGGGCTGAACACTCCAATTA -3'
	R	5'- GGCTGCTGCAAAGATTGATTAG -3'
